# A simple technique to overcome self-focusing, filamentation, supercontinuum generation, aberrations, depth dependence and waveguide interface roughness using fs laser processing

**DOI:** 10.1038/s41598-017-00589-8

**Published:** 2017-03-29

**Authors:** Jerome Lapointe, Raman Kashyap

**Affiliations:** 1 FABULAS, Department of Engineering Physics, Polytechnique Montreal, 2900 Edouard-Montpetit, Montreal H3T 1J4, Canada; 2 Department of Electrical Engineering, Polytechnique Montreal, 2900 Edouard-Montpetit, Montreal H3T 1J4, Canada; 3 Poly-Grames Research Center, 2500 Chemin Polytechnique, Montreal H3T 1J4, Canada

## Abstract

Several detrimental effects limit the use of ultrafast lasers in multi-photon processing and the direct manufacture of integrated photonics devices, not least, dispersion, aberrations, depth dependence, undesirable ablation at a surface, limited depth of writing, nonlinear optical effects such as supercontinuum generation and filamentation due to Kerr self-focusing. We show that all these effects can be significantly reduced if not eliminated using two coherent, ultrafast laser-beams through a single lens - which we call the Dual-Beam technique. Simulations and experimental measurements at the focus are used to understand how the Dual-Beam technique can mitigate these problems. The high peak laser intensity is only formed at the aberration-free tightly localised focal spot, simultaneously, suppressing unwanted nonlinear side effects for any intensity or processing depth. Therefore, we believe this simple and innovative technique makes the fs laser capable of much more at even higher intensities than previously possible, allowing applications in multi-photon processing, bio-medical imaging, laser surgery of cells, tissue and in ophthalmology, along with laser writing of waveguides.

## Introduction

Over the last decades, integrated photonics has seen among the most revolutionary advances in research. Integrated photonics chips even represent a vital part of modern society since the Internet is enabled by arrayed waveguide gratings, optical splitters and Mach–Zehnder modulators that route, split and multiplex optical signals as well as perform the conversion from electrical to optical signals in order to interface with computers. However, there is still much to be done as many of these components are still relatively expensive to manufacture. Clean room facilities, as well as several expensive manufacturing steps such as phase mask fabrication or photolithography are needed for mass production of optical devices. With its growing advancements and breakthroughs^1–4^ such as optical data storage^5, 6^, multiphoton nanofabrication^6, 7^, optofluidic lab-on-a-chip devices^8, 9^, integrated photonics devices^10, 11^, elements of optical quantum computing systems^12^, 3D photonics crystals^13^, micro-mechanical/biological systems^14, 15^, cancer treatment^16^, photonics wire bounding^17^ and invisible photonics devices for mobile phone applications^18, 19^, femtosecond laser processing is widely believed to offer a potentially versatile and cheap solution for material processing and device manufacturing. However, several limitations must be overcome to make this technology viable, namely, optical aberrations in focusing optics^20^, undesirable ablation^18, 21^, depth of field^22^, depth of writing limited by the working distance of the lens^23^, pulse stretching^24^, and unavoidable nonlinear optical effects such as supercontinuum generation and filamentation due to Kerr self-focusing^25–29^. Beam shaping has been successfully used to improve some of these problems^30^. Although a focused ring-shaped beam is well known to produce filamentation and beam breakup^31–33^, it may tighten the focal spot^34^, which has been successfully used in nanoscopy^35^ and direct femtosecond laser multi-photon polymerization^36^. Beam shaping using slits^37^ or cylindrical lenses^38^ has been used to form symmetrical waveguides. Using gratings, simultaneous spatial and temporal focusing (SSTF) reduces detrimental nonlinear interactions^39^. Unfortunately, all the problems cannot be solved simultaneously, as different techniques only solve a single issue at a time.

In this article, we present a simple fs-laser processing method which improves, simultaneously, all the aforementioned limitations. As a main result, the new technique drastically suppresses supercontinuum generation and extended filament formation, which are detrimental effects that limit the precision of laser processing, e.g., nano-structuring and in cell, tissue^40^ and ophthalmic^41^ fs-laser surgery. The new technique that we refer to as the Dual-Beam (DB) technique uses two parallel beams focused together through a single lens. The method along with the new parameters will be first explained. Analysis and results demonstrating significant improvements of each of the problems mentioned above is then presented. Finally, an extended discussion explains further, the potential uses of this new technique and discusses the drawbacks with proposed solutions.

### The Dual-Beam parameters

**Figure 1:**
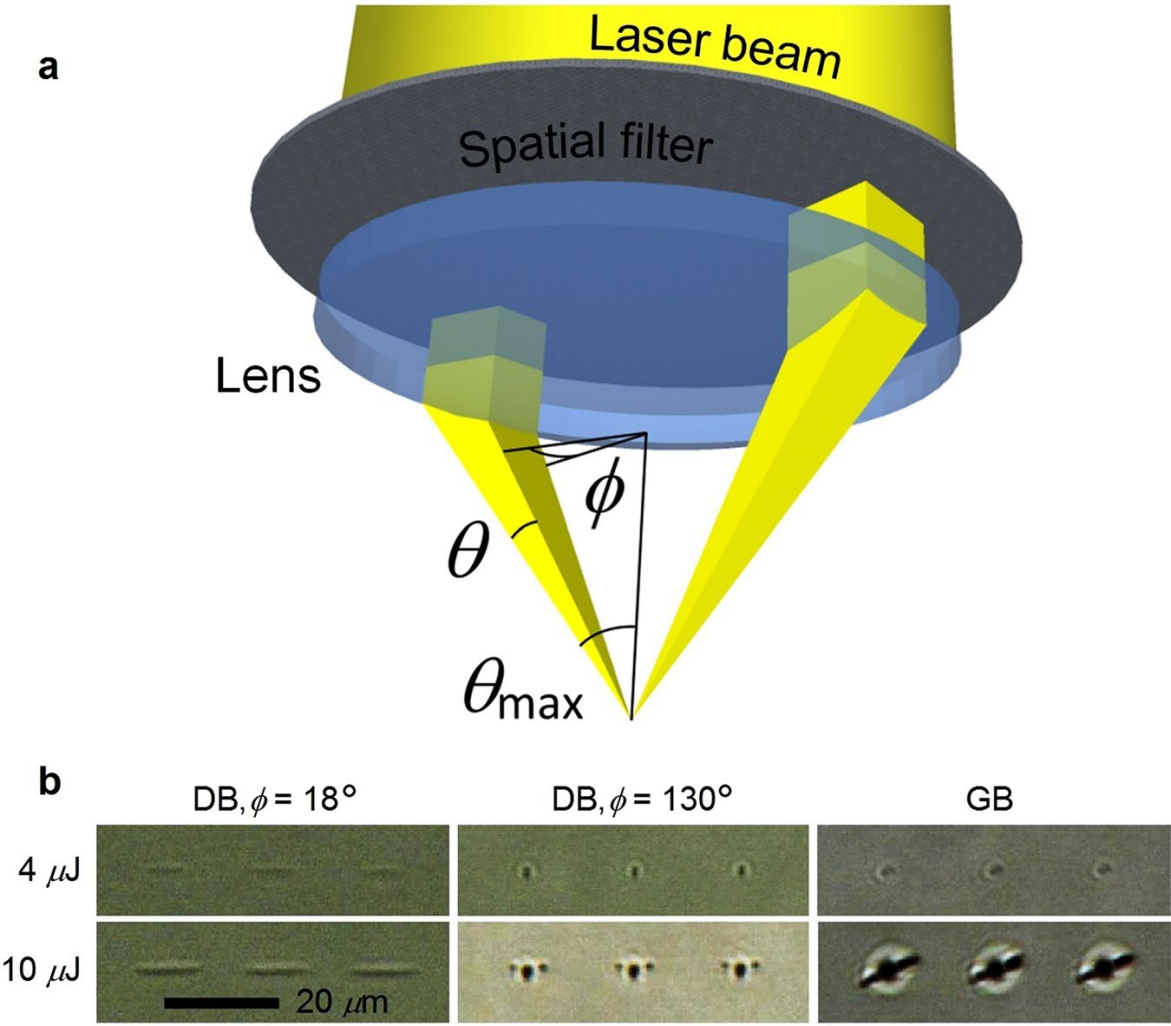
The Dual-Beam laser processing scheme: (**a**) focusing two parallel laser beams formed with a spatially filtered Gaussian beam, using two holes in a metal plate. (**b**) The Dual-Beam (DB) method tightens the laser induced structural modification spots (in one axis for low *ϕ* and in the two axes for high *ϕ* values) compared to a conventional Gaussian beam (GB) focusing. Each picture represents the top view of three fs-laser spots in glass for pulse energies of 4 and 10 *μ*J at 1030 nm wavelength focused with a 0.55 NA lens.

The purpose of this processing method is to obtain two identical pulsed laser beams, parallel and coherent, separated by a distance equal to the diameter of a focusing lens in order to preserve the maximum numerical aperture (NA). As shown in Fig. 1a, a spatial filter with two holes was used in this work to obtain the DB to overlap at the focus. Other methods to form the DB, which use a higher proportion of the laser intensity, are described in the Methods section. Note that every power or energy value mentioned in this article is measured at the focus, i.e. after the spatial filter and the lens.

Figure 1a shows the two major parameters of this new laser processing method: *θ*, which represents the portion of the NA used (NA = sin *θ*_max_) and *ϕ* is the arc of the ring, i.e. the circular portion of the beam (the spatially unfiltered beam has *ϕ* = 180° and *θ* = *θ*_max_). A pixelated liquid crystal modulator can be placed before the lens to easily vary *ϕ* and *θ* ^42, 43^. Note that the plane formed by the two parallel focused beams must be perpendicular to the laser writing direction, since the shape of the focal spot is elliptical.

### Pulse stretching and tight focusing for laser nanoprocessing

Nano-processing and nanoscopy via multi-photon absorption is now achievable using ultrashort laser pulses tightly focused into material^6, 7, 35^. Hollow beams have been shown to generate slightly tighter focal spot size compared to Gaussian beams^34^. As this new Dual-Beam method is based on a portion of a hollow beam, the tightening effect is also seen (see Fig. 1b). For low *ϕ* values, the tightening effect is seen along one of the two axes perpendicular to the lens axis, whereas the focal spot size along the other axis is elongated (see Fig. 1b with *ϕ* = 18°). By increasing the *ϕ* value, the spot size reduction quickly affects the other axis since the NA increases, thus the laser spot shape becomes circular (see Fig. 1b with *ϕ* = 130°). The affected zone elongation can be widely controlled using different values of *ϕ*, which effectively changes the NA parallel to the scan direction, while the thinness can be slightly changed using different values of *θ*. Therefore, the DB method with low *ϕ* values suits single-dimensional processing applications very well, such as microfluidic, waveguide based photonics devices or cutting tissues with fs-surgery, whereas a circular laser spot shape using high *ϕ* values suits nano-structuring, optical memory storage or cell treatment, for example. Note that using even the maximum energy pulses available with our laser that can reach the glass (20 *μ*J), the DB method with *ϕ* = 18° does not generate cracks as seen with the use of circular laser spots made using 10 *μ*J pulses (see the dark lines beside the central spots in Fig. 1b, bottom right). This can be explained by the fact that the heat and stress is better dissipated from an elongated shape. However, cracks should be seen using higher laser intensity even using the DB technique with low *ϕ* values. Note that under a high repetition rate these cracks can melt and disappear^44^.

It is well-known that the pulse width can limit the minimum size of the laser processed area^45^. Lens aberrations stretch a laser pulse partly due to propagation time difference (PTD) and group velocity dispersion (GVD) (see Methods and ref. 24). Multi lens system such as plan achromatic objectives can strongly reduce these aberrations but do not solve aberrations from the refractive index mismatch between the sample and immersion medium, especially for deep processing. Nevertheless, these aberrations can be compensated for using a spatial light modulator (SLM) with the conjugate phase pattern^22, 23, 46, 47^. However the efficiency is limited by the SLM resolution and the phase pattern must be tuned for different wavelengths, lens NA and depth of writing. This poses some difficulties but can be accomplished by sensing the plasma emission during the inscription process and using a feedback loop to optimize the plasma brightness^46^.

In the new DB laser processing technique, as the two beams are launched at an identical radial distance (the maximum radius *r*_0_) from the lens’ optical axis, spherical aberrations from lenses and refractive index mismatch, as well as temporal delays due to GVD and PTD are strongly reduced. To demonstrate this phenomena, a laser beam with an initial pulse duration *τ*_p_ = 420 fs was launched into two 40× lenses (NA = 0.55 and radius *r*_0_ = 2.5 mm) with a common focus so that the beam is focused and brought back to being parallel in order to be measured. As the lenses were designed for 780 nm wavelength, broadening is expected since the wavelength used is 1060 nm. A 140 fs broadening from PDT and 5 fs due to GVD are also calculated (see Methods).

**Figure 2:**
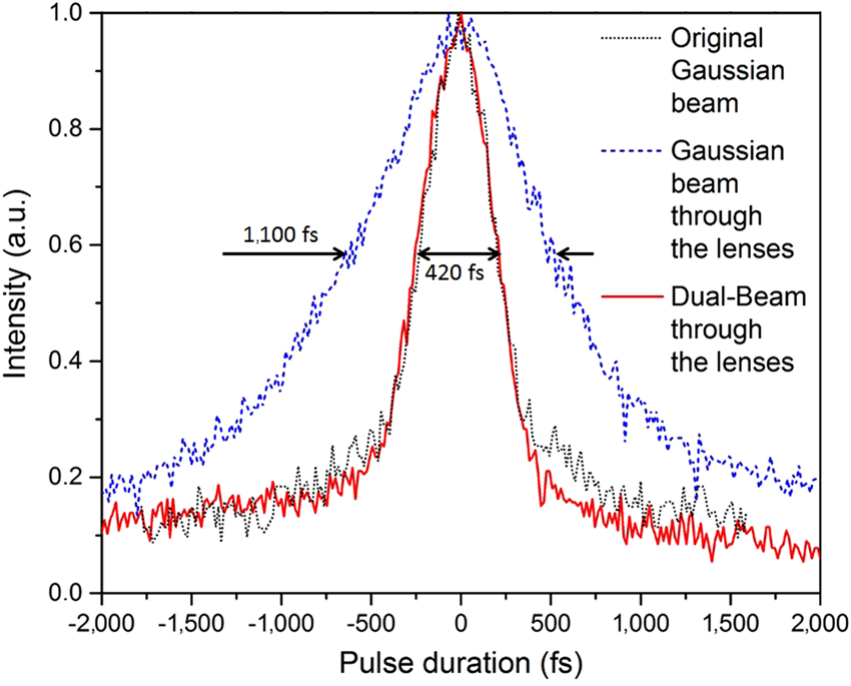
The ultrashort laser pulse duration remains unaffected after been focused using the Dual-Beam technique compared to conventional focusing.

Figure 2 shows the pulse broadening difference through the lens system between the original Gaussian beam and the DB (*ϕ* = 30° and *θ* = 5°), measured using an auto-correlator. The two lenses broadened the pulse from the original Gaussian beam to over 600 fs, while the pulse does not seem to be affected using the DB technique. Since the pulse remains ultrashort using the DB technique, increased detrimental nonlinear effects such as supercontinuum generation and filamentation should be expected. Fortunately, as demonstrated in the next section, the DB technique eliminates most detrimental nonlinear effects due to its cross focusing shape.

Note that, for the case shown in Fig. 2, the pulse broadening Δ*τ* due to GVD is almost negligible since it is inversely proportional to the pulse duration *τ*_p_ (see Methods eq. (2)). Therefore, a sub-femtosecond pulse may generate pulse broadening in the picosecond range. Since attosecond laser applications are increasing^48^, it would be interesting to validate the efficiency of the DB technique to keep the short pulse duration in such cases. Note that the SLM technique mentioned earlier can be added to the DB technique for further efficiency.

### Limiting detrimental ablation, supercontinuum generation and filamentation

Pulses with peak power over a certain value can trigger Kerr induced strong self-focusing and self-phase modulation. These nonlinear effects are damaging since they reduce the precision of the applied modifications and induce side-effects such as strong supercontinuum generation (SCG), produced by nonlinear frequency conversion, self-steeping of the pulse and sharp plasma gradients^49^. Moreover, the complex interaction of Kerr induced self-focusing and the defocusing refractive index change of the induced plasma can result in the creation of detrimental filaments^25–29^. Recently, simultaneous spatial and temporal focusing (SSTF) was proposed to overcome these limitations^39^. The SSTF method uses gratings to spatially disperse the spectral components of a broadband ultrashort laser pulse outside of the focal spot, which results in a temporal pulse stretching. At the focus, the entire pulse spectrum superposes locally and compresses the pulse to its bandwidth-limited pulse duration. Because the ultrashort pulse duration is restricted to the focal spot, the out of focus intensity is strongly reduced, thereby reducing undesirable pulse-material interactions.

**Figure 3:**
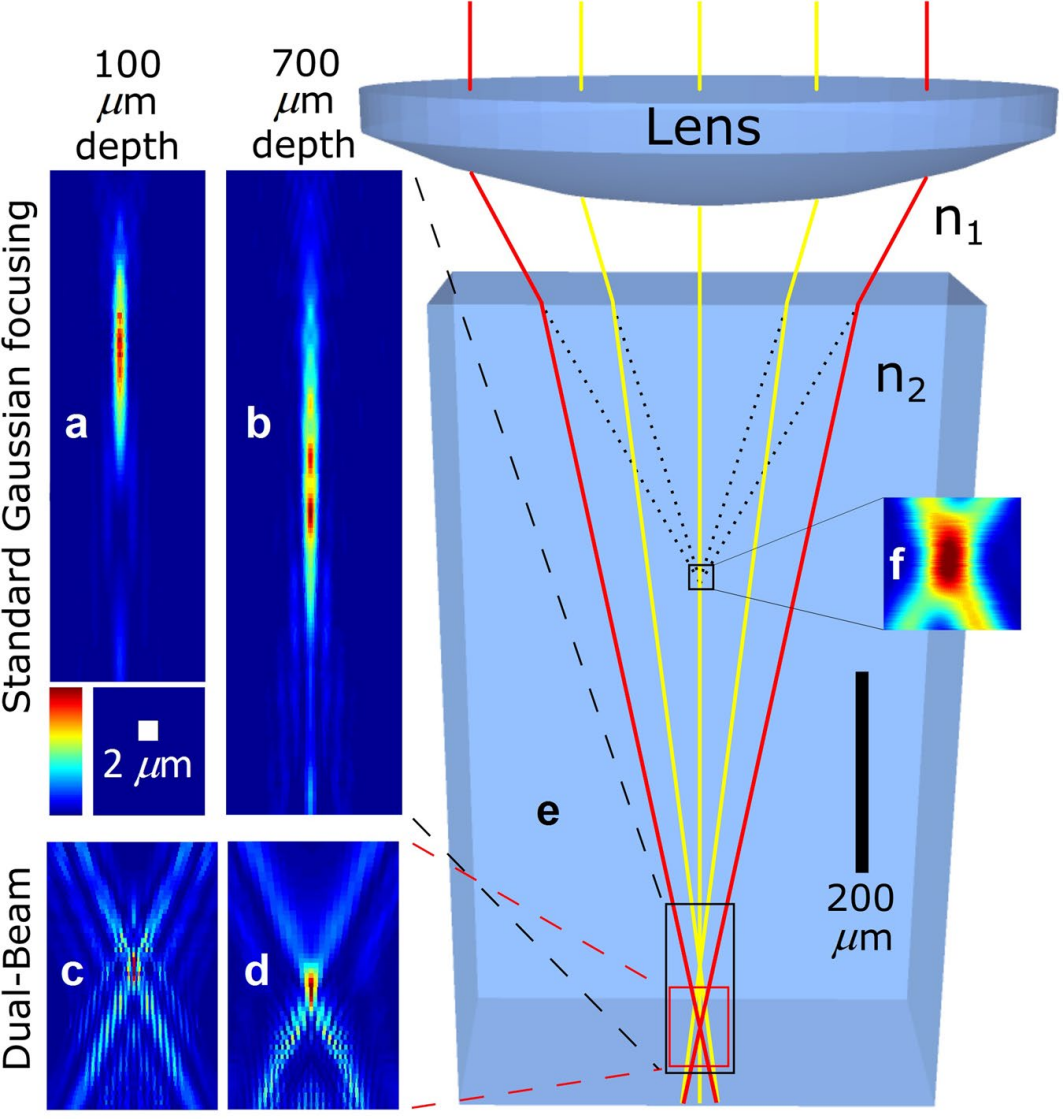
The Dual-Beam technique strongly reduces the spatial aberration at the focus. The simulated intensity profile of the focal spot is elongated when focused in a sample using a standard focusing method (at 100 *μ*m depth, (**a**), and at 700 *μ*m depth, (**b**)) while it remains similar using the Dual-Beam technique with *ϕ* = 19°, *θ* = 4° ((**c**) and (**d**)). (**e**) The colored Zemax simulation shows the ray tracing in the sample (*n*_2_ = 2.4) focused with a 0.55 NA lens at a depth of 700 *μ*m. The red rays represent the Dual-Beam technique. (**f**) Experimental measurement of the focus intensity profile in air *n*_1_ = 1 (see Methods for more information). The 2 *μ*m scale bar is valid for (**a**) to (**d**).

With the DB method on the other hand, there is no need for any additional measures; the high intensity occurs only at the focal spot. In fact, since each of the two beam diameters is initially very small before the lens, they are both focused at very low NA (with low energy density) until they meet to form a high NA at the focus. To generate a filament, a minimum intensity is needed to trigger the process beyond the focal spot along the optical axis, which is accessible from a focused un-spatially filtered Gaussian beam (see Fig. 3a and b), focused at 100 and 700 *μ*m, respectively, under the sample surface). However, with the DB method, a high laser intensity is no longer available beyond the focal spot (see Fig. 3c and d, also focused at 100 and 700 *μ*m, respectively). Note that Fig. 3b and d are zoomed portions of the colored Zemax ray tracing simulation in Fig. 3e, which shows the intersections of the rays at 700 *μ*m depth. Moreover, the *k*-vectors prevent filamentation under the focal spot since momentum is not conserved. Even using our highest pulse energy available that can reach the glass (20 *μ*J), no filamentation was observed in standard glass using the DB method, whereas a pulse energy of few *μ*J can generate extended filaments^28^.

**Figure 4:**
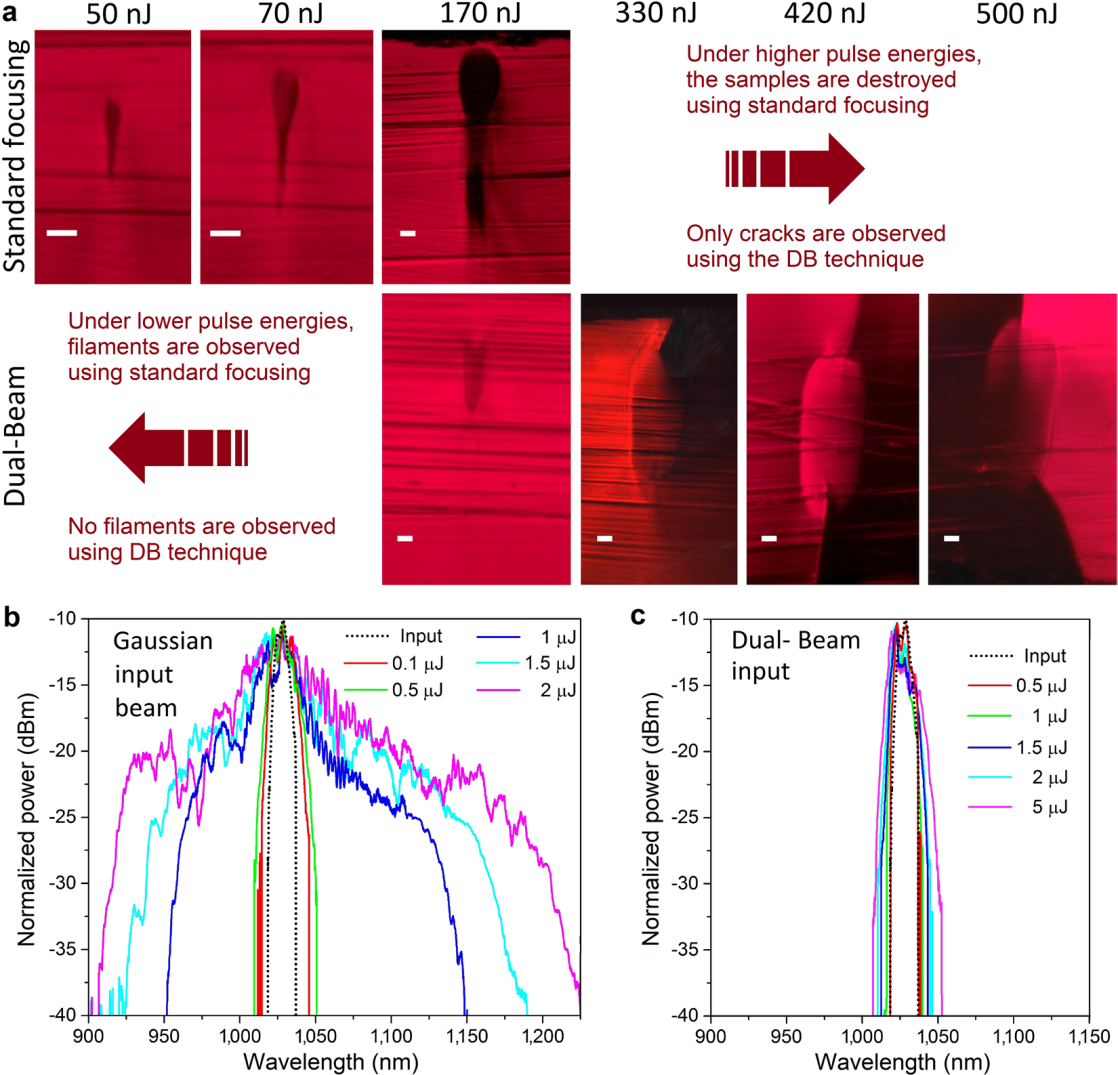
The Dual-Beam technique strongly reduces the detrimental nonlinear effects. (**a**) filaments are formed in chalcogenide glass under low pulse energy using the standard Gaussian focusing method while none are formed up to 330 nJ pulse energy (the scale bars are 5 *μ*m). (**b** and **c**) Spectral response of a 1030 nm laser (doted black line) focused through a chalcogenide sample. The Dual-Beam laser processing technique (**c**) strongly reduces supercontinuum generation compared to conventional focusing (**b**).

In order to favour filamentation using the DB technique, chalcogenide glass (a highly nonlinear material) was used with large values of *ϕ* = 130° and *θ* = 12°. Lines were written using a 1030 nm fs-laser at a repetition rate of 600 kHz, a scan speed of 10 mm/s and using a 0.55 NA lens. Figure 4a shows the facet views of the filaments formed under conventional Gaussian focusing with pulse energies as low as 50, 70 and 170 nJ, whereas no filament was noticed up to 330 nJ using the DB technique. Moreover, for higher pulse energies, it is difficult to determine if the lines under the focal spot come from filamentation or from the stress that gives rise to a crack through the full thickness of the sample (see Fig. 4a, bottom right).

Since SCG is proportional to the nonlinear interaction length, no strong supercontinuum can be generated with the DB technique. In order to compare SCG from the DB technique with conventional focusing, chalcogenide glass was used, since it has a large nonlinear coefficient. Figure 4b and c show the SCG broadening measured using an optical spectrum analyzer (OSA) from a 1030 nm wavelength input fs-laser with pulse energy from 0.1 to 2 *μ*J at a repetition rate of 50 kHz focused with a 0.65 NA lens for each of the laser inscription methods. Figure 4b shows how the DB technique strongly reduces the SCG even using our spatial filter with the closest parameters (*ϕ* = 130° and *θ* = 10°) to a full beam for this lens. Moreover, even with a 5 *μ*J pulse very weak SCG is noticed. Note that the 250 *μ*m thick chalcogenide sample broke using the 5 *μ*J pulse with the conventional Gaussian input beam.

These results are of high interest for cells, tissues^40^ and ophthalmic^41^ fs-surgery. In fact, the increased breakdown length due to the nonlinear side effects reduces the quality of surgical cuts and harms tissue outside the focal spot, while extended disrupted cut patterns decrease the visual quality of the eye^41^. Moreover, retinal safety limits may be affected due to the broadband SCG^39^. Since the biological tissue consists primarily of water, it would be of great interest to replicate the work of Kammel *et al*.^39^ to test the DB technique’s efficiency to limit the nonlinear side effects in water. Note that it would be also very interesting to combine the SSTF method^39^ with the DB technique. We believe that this combination should further prevent the nonlinear side effects.

Nonlinear absorption generates undesirable ablation when the laser is focused close to the surface of a glass sample, which is restrictive for certain applications such as compact multi-layers, lab-on-a-chip, waveguide based evanescent wave surface sensor or flexible thin glass processing. Recently, an efficient and simple solution was demonstrated by Bérubé *et al*.^21^ using a 150 *μ*m thick glass cover slide placed in optical contact with the top surface of the sample to be processed. The sample is thus virtually thicker and the sample can be laser processed at the very edge of the surface (see Methods for more information and limitation of this technique). Waveguides have also been written close to the surface in toughened glass such as Corning® Gorilla® glass^18, 19, 21^ in which ion exchange process forms a compressive layer with high mechanical resistance that limits cracks and surface ablation. Since Gorilla glass is used as screen covers in the display industry, it has a great potential for liquid and gas sensing applications in mobile devices^19^.

**Figure 5:**
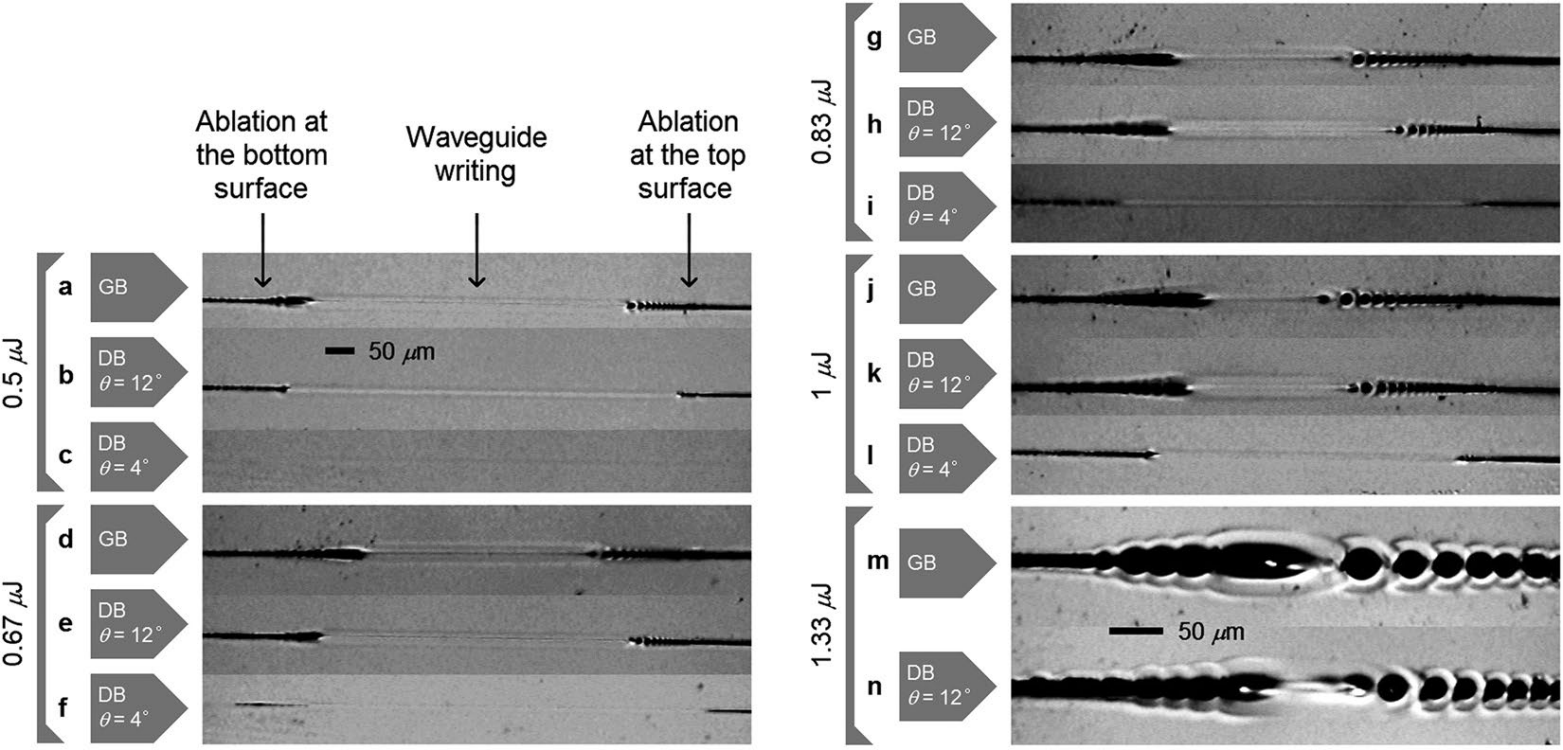
The DB technique limits detrimental surface ablation. Top view of diagonally laser written lines from the top to the bottom surface of a 100 *μ*m thick flexible glass sheet. The results show that using the Dual-Beam (DB) technique with *θ* = 12° (**b**,**e**,**h**,**k** and **n**) and *θ* = 4° (**c**,**f**,**i** and **l**) it is possible to write waveguides closer to the glass surface compared to the conventional Gaussian beam (GB) focusing (**a**,**d**,**g**,**j** and **m**). The laser was focused with 0.5 *μ*J pulses (**a**,**b** and **c**), 0.67 *μ*J pulses (**d**,**e** and **f**), 0.83 *μ*J (**g**,**h** and **i**), 1 *μ*J (**j**,**k** and **l**) and 1.33 *μ*J (**m** and **n**). The 50 *μ*m scale in (**b**) is the same from (**a**) to (**l**) and the scale in (**m**) is also valid for (**n**). The contrast has been enhanced to make the features visible.

Using the DB method without any additional measures or preparation, structural modification closer to a sample surface can be realized compared to conventional focusing. Figure 5 shows a comparison between conventional Gaussian focusing (a, d, g, j and m), the DB method with *θ* = 12° (b, e, h, k and n) and the DB method with *θ* = 4° (c, f, i and l). In each case, fs-laser writing with a 6% diagonal slope was used from the top to the bottom of a 100 *μ*m thick glass sheet. The 1030 nm wavelength fs-laser with a repetition rate of 600 kHz was focused using a 0.55 NA lens. For the DB cases, *ϕ* = 130° was chosen in order to obtain high enough power. The comparison was made using pulse energy from 0.5 *μ*J to 1.33 *μ*J. It is only possible to write waveguides through a height of 48 *μ*m over the total glass thickness using conventional focusing with 0.67 *μ*J pulses (Fig. 5a), and through 62 *μ*m using the DB technique with *θ* = 12° (Fig. 5b), which is a good improvement. Only some micro-roughness (no ablation visible to the naked eye) is created using the DB technique with *θ* = 4° (Fig. 5c). With 1.33 *μ*J pulses, it is impossible to write waveguides using conventional focusing (Fig. 5m) while it is possible using the DB method with *θ* = 12° (Fig. 5n). Note that not enough laser power was available with our laser to test the DB technique with *θ* = 4°. See Methods for more information on waveguide writing in flexible thin glass.

**Figure 6:**
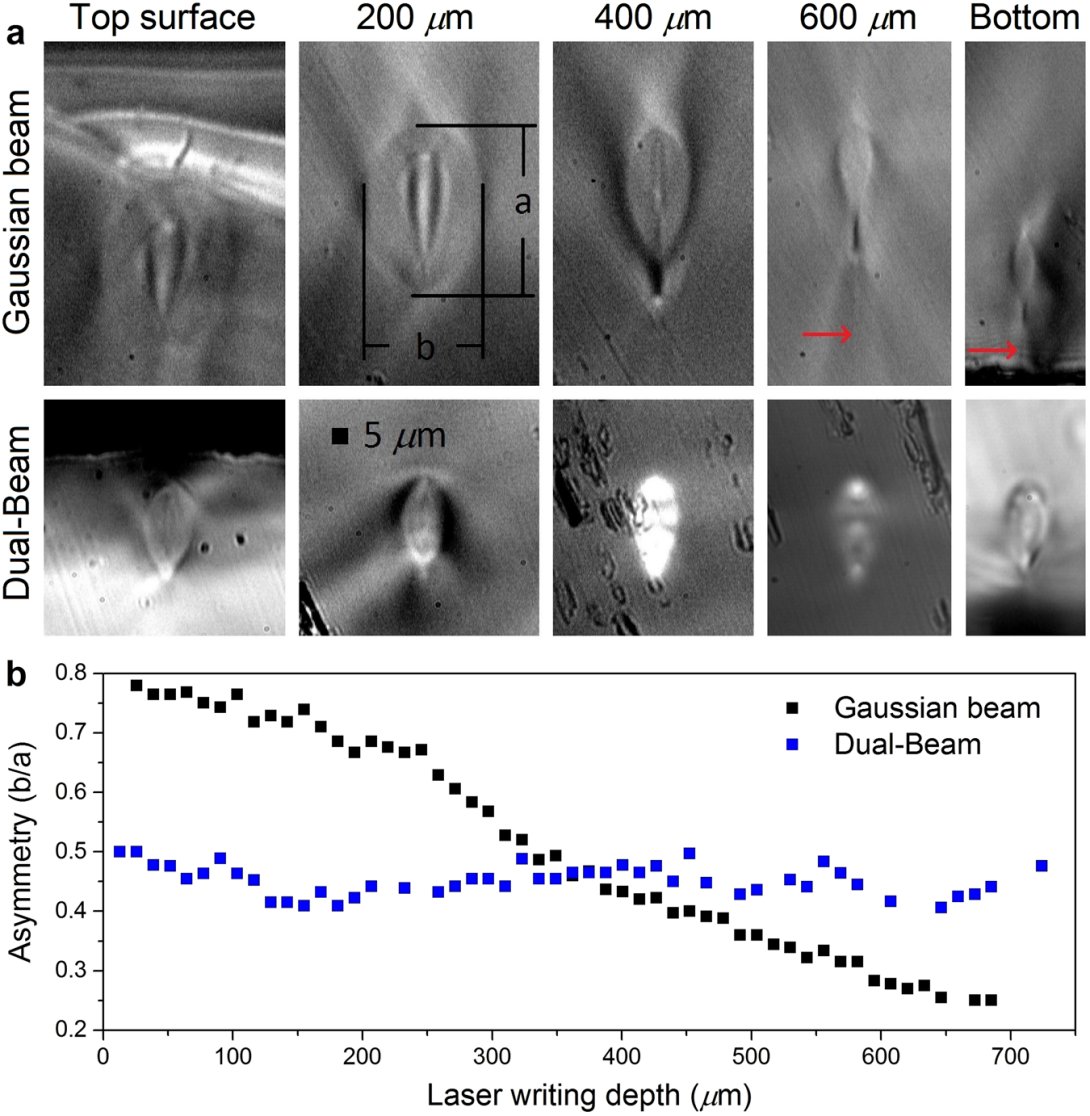
The laser writing depth dependency is strongly reduced using the Dual-Beam technique compared to the conventional focusing technique. (**a**) Facet view of the waveguides at different depths. Note that the red arrows show weak filaments produced under low energy pulses (1 *μ*J) as discussed earlier. (**b**) Graph of the measured asymmetry showing the depth of writing dependency.

### Depth dependency and optical aberrations

Aberrations are focusing depth dependent and reduce the focal intensity, thus limiting the 3D capability of ultrafast laser inscription. As simulated (with a 0.55 NA lens, *ϕ* = 18°, *θ* = 4° and a high refractive index material *n*_2_ = 2.4 to simulate the worst case) in Fig. 3a to e and demonstrated experimentally in Fig. 6 (with a 0.55 NA lens, *ϕ* = 18°, *θ* = 25°, focused into Corning Gorilla glass with *n*_2_ = 1.51), the focal spot is vertically elongated proportionally to the depth in the material using conventional Gaussian focusing, whereas no elongation can be observed using the DB technique. Figure 6b shows the waveguide asymmetry *b*/*a* (waveguide cross section width *b* over its height *a*) as a function of the laser writing depth. The DB technique clearly demonstrates an improvement over the conventional focusing method. Note that we have obtained similar results in soda lime, BK7, Corning Eagle glasses and quartz. Since coupling efficiency is dependent on the waveguide geometry, the DB technique could standardize a coupling method with no depth dependence. These results give rise to significant advantages for standardizing the laser writing recipes for multilayer devices, which we believe to be helpful in meeting the criteria required for mass deployment of future photonics devices. Moreover, this depth independence can be useful for opto-fluidics applications where the micro-channel may be limited to an elongated shape.

Symmetrical waveguides are usually preferred principally due to the higher light coupling efficiency from standard optical fibers which are perfectly symmetric. As shown by the simulations in Fig. 3, the focal spot symmetry is greatly improved using the DB technique with small value of *θ* (the simulation used a *θ* = 4°). For large values of *θ*, it has been demonstrated experimentally (with *θ* = 25°) that the waveguide is more symmetric only for deep focusing (see Fig. 6), but cannot be perfectly symmetrical. It would be of great interest to test lower values of *θ* experimentally in order to obtain the symmetrical limit. Using conventional Gaussian focusing, it has been shown that beam shaping using a slit^37^ or cylindrical lenses^38^ placed just before the focusing lens makes the waveguides more symmetric. However, those methods cannot be added to the DB technique to further improve symmetry since the beam is already precisely shaped. Note that depth dependence is also reduced by combining the slit method and the use of an SLM^22^ but remains for deep (>1 mm) laser machining^47^.

### Optical propagation loss

**Figure 7:**
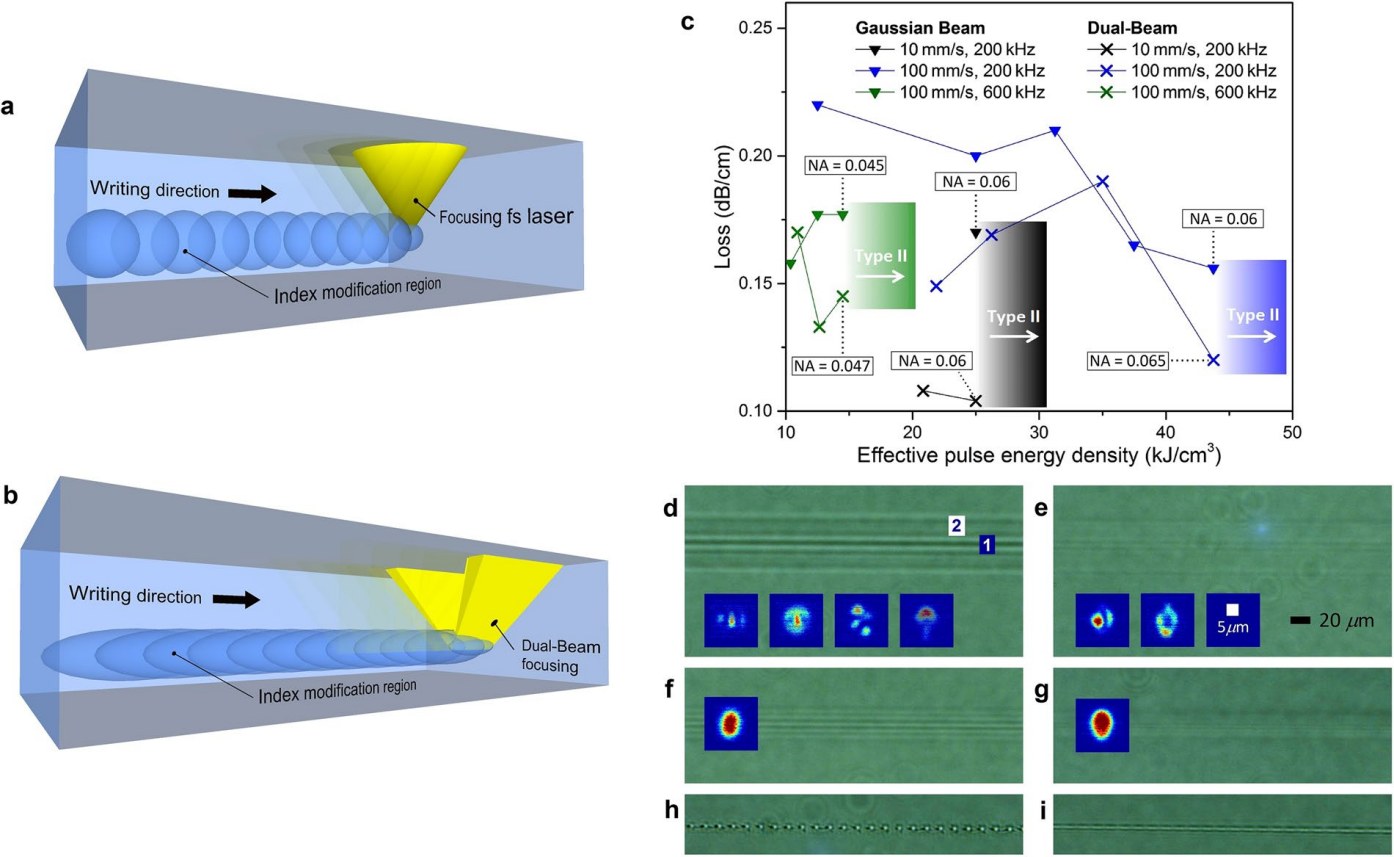
Lowering the propagation loss due to the waveguide interface softening using the DB technique. (**a**) Waveguide writing scheme using conventional fs-laser focusing. (**b**) Waveguide writing scheme using the Dual-Beam technique showing the softening of the waveguide interface. (**c**) Waveguide propagation loss comparison between the conventional Gaussian beam laser writing technique (triangles) and the Dual-Beam technique (×) with *ϕ* = 18° and *θ* = 25°. The three different colors represent three different recipes. Waveguide top views (**d** to **g**) and their respective near field mode profiles (inset images). Multimode (**d** and **e**) and single-mode (**f** and **g**) waveguides fabricated using the conventional Gaussian focusing method (**d** and **f**) and using the DB technique (**e** and **g**). The delimitation between the waveguide zone (**d1**) and the heat affected zone (**d2**) is more visible with the Gaussian technique. When the writing spots are too far apart, it is impossible to write functional waveguides using conventional focusing (**h**) whereas it is possible using the Dual-Beam technique (**i**) due to the elongated spots. Note that the error bars (±0.03) obtained from the four measurements on every waveguide propagation loss, are not shown in the graph (**c**) in order to simplify the figure.

Since the first glass laser written waveguides in 1996 by Davis *et al*.^50^, the lowest recorded propagation loss was reported to be 0.1 dB/cm, which was far from the propagation losses < 0.008 dB/cm readily achieved in silica-on-silicon in 1994^51^. Nevertheless, since the new hypothesis proposing that significant loss originates from laser induced waveguide interface roughness (see Fig. 7a) rather than local defects, the lowest recorded loss up to date has been reached: 0.05 dB/cm using annealing^52^ and recently 0.027 dB/cm using the stress in ion-exchanged processed toughened glass^18^. Such propagation losses start to be of importance for integrated devices and lab-on-a-chip applications. Here we show that smoothing the waveguide interface is also achieved directly using the DB technique. As shown in Fig. 7b, the refractive index modification region induced by one laser pulse is elongated parallel to the waveguide which reduces the waveguide roughness. The lower the *ϕ*, the higher will be the elongation. Due to the spherical shape at the focus of a Gaussian beam (see Fig. 7b to the right) or a ring-shaped beam^53^, waveguide roughness is produced while laser writing. To demonstrate this phenomenon, the DB technique was compared to conventional waveguide writing in Corning Eagle glass. Fig. 7c shows preliminary results with a few writing recipes of type I waveguides (in which the waveguide is created at the focus). Scan speeds of 10 and 100 mm/s, repetition rates of 200 and 600 kHz, and pulse energies from 0.75 to 10 *μ*J were used. For all the waveguides, a 1030 nm wavelength fs-laser was focused using a 0.55 NA lens. The overall tendency shows that the DB technique produces lower loss waveguides. Note that since the focal spot shapes of the two writing techniques differ, an “effective pulse energy density” is used as the ×-axis in Fig. 7c (see Methods).

The well-known cut-back method was used to measure the propagation loss of every waveguide using a 29 cm long glass plate cut to a 12 cm long piece. The overall lowest loss type I waveguide (∼0.1 dB/cm at 1550 nm) was achieved using the DB technique with the following parameters: 10 mm/s scan speed, 200 kHz repetition rate and pulse energy of 3 *μ*J. The top view of the few-mode waveguide and its corresponding near-field mode profiles are shown in Fig. 7e. The corresponding waveguide using conventional focusing (see Fig. 7d) shows a propagation loss of 0.17 dB/cm and seems to support few more modes probably due to its slightly larger size. The near-field mode sizes (∼10 *μ*m) suggest that the light is guided in the central refractive index change zone (Fig. 7d1), which can be distinguished from the heat affected zone in Fig. 7d2. The waveguide zone delimitation is clearly more visible in the conventional focusing technique (Fig. 7d and f) which supports the hypothesis that the DB technique (Fig. 7e,g and i) softens the waveguide interface. The single-mode waveguide top views from the 100 mm/s scan speed and 600 kHz repetition rate recipe are shown in Fig. 7f for the conventional focusing method (with a minimum propagation loss of 0.16 dB/cm) and Fig. 7g for the DB method (with a minimum propagation loss of 0.13 dB/cm). The near-field single-mode profiles from the conventional focusing and the DB technique seem very similar. The lower loss difference between the two latter waveguides is probably resulting from the fundamental mode interacting less with the waveguide interface.

Moreover, due to the elongated focal spot induced by the DB technique, it is possible to write waveguides when it was not possible with standard focusing. For certain scan speed and repetition rate combinations (see the example of 10 mm/s and 1 kHz in Fig. 7h and i) a continuous waveguide is only possible with the DB technique. The recipes with 10 mm/s scan speed combined with 1 kHz repetition rate or 100 mm/s with 10 kHz, with pulse energy from 1 to 10 *μ*J resulted in functional waveguides only when using the DB method. This phenomenon may be of interest for waveguide Bragg grating applications since the pulses form a periodic refractive index change in the waveguide^54^.

Note that these tests are only preliminary results which prove the lower propagating loss using the DB method. However, it would be interesting to test more parameters (wavelength, repetition rate, pulse width, focusing lens NA, power, scan speed, number of scans, polarization) in order to find the lowest loss recipe for every type of glass.

## Discussion

Waveguide NA is also an important parameter. Tight bends for compact integrated devices require high NA while low NA is required to produce invisible devices for display screen applications which have recently gained high interest since the first invisible devices were demonstrated in smartphone screens which include thermometer^18^ and refractive index sensor^19^. No significant NA changes were observed between the two focusing techniques. The NA was measured using the well-known formula NA = *sin* *θ*, where *θ* is the maximum angle (at 5% of the maximum intensity) of the far-field of the mode from the waveguide axis. The NA measurement method was successfully tested using a standard Corning® SMF-28® fiber. Some results are shown in Fig. 7c with an error of ± 0.005. In general, a slightly higher NA is observed for the DB technique probably due to the softened waveguide interfaces which propagate better the higher modes which are usually excited under higher NA. However, more tests and statistical studies should be realized to support this hypothesis.

High quality waveguides with sub-millimetre radius bends are not possible yet using laser writing due to the limited waveguide NA produced. However, even if high NA waveguide fabrication becomes possible using direct laser writing, high quality waveguides with very tight bends can probably not be produced using the DB laser writing technique. In fact, as shown in Fig. 1b, the elongated laser spots using low *ϕ* values would form a waveguide interface roughness in a very tight curve. Using the laser spot shape with *ϕ* = 18° (see Fig. 1b), curved waveguides were simulated and we found that the DB technique would start inducing waveguide interface roughness only with curve radius below 100 *μ*m. Nevertheless, the laser writing method could be switched to a conventional writing method while writing a curve. To better maintain a similar waveguide shape, the laser writing method should rather be switched to a hollow beam (*ϕ* = 180°). From the hollow beam, a motorized slit could be used simply to vary the *ϕ* parameter. Note that this slit must always be perpendicular to the waveguide thus also be motorized angularly. An even simpler solution would be to use pixelated liquid crystal modulator^42, 43^ synced with the writing direction.

For certain applications, the DB technique parameters *ϕ* and *θ* should be as small as possible. However, there is a diffraction limit. In fact, if *ϕ* and *θ* are chosen to form micron size beams, both beams would diverge and the focal spot would enlarge. It would be of great interest to study the optimal *ϕ* and *θ* values for every laser processing applications. Unfortunately, the laser power in our laser was not enough to perform this study using a spatial filter similar to that shown in Fig. 1a. In fact, larger values of *ϕ* and *θ* have been chosen in this work in order to obtain enough power to prove our hypotheses.

High precision nanoscopy and nanoprocessing require high NA lenses. However, such lenses have a short working distance which limits the processing depth. In fact, high NA lenses with large working distance require large optical elements, which are expensive and very complicated to fabricate. The absolute NA precision and the relative NAs that have to be matched perfectly at every lens radius are two criteria difficult to meet. For most applications, the absolute NA precision is not crucial. As an example, for an application that requires a 0.55 NA lens, a 0.5 or 0.6 NA lens would probably do the job. On the other hand, to obtain an optimal focal spot, perfectly matched relative NAs at every radius of the lens are essential and very complicated to fabricate for large lenses. As opposed to the conventional focusing, the DB technique does not use the full area of the lens; only a small part is needed at the edge of the lens. Therefore, only two identical small pieces of glass are needed, which could be simpler to fabricate. These two glass pieces could be made to be used far from the material being processed and thus increase the processing depth. This is also of great interest for longitudinal waveguide writing which most researchers have abandoned due to the short working distance of the lenses limiting the waveguide length to ∼3 mm^23^.

Finally, note that the DB technique is not limited to two beams alone but can use multiple-beams focused together through a single lens, depending on the application and the spot shape required at the focus. For example, four beams equidistant from the lens axis form a cross shaped focal spot, i.e. two elongated spots like the spots shown in Fig. 1b with *ϕ* = 18°, superposed perpendicularly. The tight spot made at the overlapping region, has twice the intensity, and can be useful for precise polymerisation and structuring.

## Conclusion

In this work, a new Dual-Beam technique has been introduced, which improves in many ways laser processing of materials. The fact that the Dual-Beam technique uses only a portion of the focusing lens, i.e. a single radius from the lens’ optical axis, implies that several laser processing problems are strongly improved. First, optical aberrations due to the refractive index mismatch at the sample interface is radius dependent, which modifies the focal spot shape as a function of the processing depth. Using the Dual-Beam technique, the focal shape remains practically unchanged for any depth. This is a great advantage to standardize the light coupling method and laser writing recipes for multilayer devices, which we believe to be helpful in meeting the criteria required for mass deployment of future photonics devices. Secondly, since most optical aberrations and temporal delays due to GVD and PTD from a lens are radius dependent, the Dual-Beam technique strongly reduces the temporal stretching of the laser pulse. A 420 fs-laser pulse launched through a 0.55 NA lens remains practically unaffected using the Dual-Beam technique while a standard focusing technique stretches the pulse by over 300 fs. Maintaining the short duration of a laser pulse while focusing is essential to bypass the nanoscale limits of laser processing. It has also been demonstrated that the Dual-Beam technique reduces undesirable ablation while processing within close distance to a sample’s surface, which is useful for certain applications such as compact multi-layers lab-on-a-chip, waveguide based evanescent wave surface sensor or flexible thin glass processing. Moreover, detrimental optical nonlinear effects such as supercontinuum generation and filamentation due to Kerr induced self-focusing are also strongly reduced principally due to the short nonlinear interaction length formed at the focal spot using the Dual-Beam technique. Even using our highest pulse energy available (20 *μ*J), no filamentation was noticed in glass using the Dual-Beam method, whereas pulse energy of only a few *μ*J formed extended filaments using conventional focusing. It has also been demonstrated that the waveguide interfaces are softened using the Dual-Beam technique due to the elongated refractive index modification region parallel to the waveguide. This smoothing results in an improvement of the waveguide propagation loss. Finally, since the Dual-Beam technique uses only two small portions of the focusing lens, the lens fabrication may be simplified. Moreover, it is believed that the two required small pieces of lens can be readily fabricated in order to increase the working distance, which would increase the laser processing depth limit and be useful for longitudinal laser writing. Indeed, we have only just scratched the surface of the potential of our new Dual-Beam laser processing technique which offers great opportunities for research and development of laser processed lab-on-a-chip applications and future photonics devices.

## Methods

### The two parallel beams formation

To obtain the Dual-Beam and conserve a higher proportion of the laser power, one may think of using a slit that cuts a ring-shaped beam (also called donut, doughnut, vortex or hollow beam) generated by a spatial light modulator (SLM)^55^, axicon, fork phase mask or spiral phase plate^56–58^. However, these methods affect the phase uniformity of the beam, which generates interference patterns at the focus, and do not generate the appropriate ring-shape required (with a low *θ* value, see Fig. 1a) due to infilling of the ring. Moreover, the loss of power that occurs generating any of these beams is still significant.

A way to obtain the Dual-Beam without losing a significant amount of laser power would be to split the initial laser beam into two using a 50/50 beam splitter cube and a path delay for accurate temporal superposition of the pulses. The two beams can be focused into a nonlinear crystal and the maximum intensity of the generated harmonic will yield a perfect pulse superposition, as is the working principle of standard auto-correlators used to measure pulse widths. Beam combining of laser arrays with high efficiency and good beam quality has been also widely studied^59–61^. Using this method, however, the phase must be carefully controlled.

Finally, a custom-made laser could be made to select the TEM01 or TEM10 mode to obtain two lobed intensity profile^62^. Therefore, the two peak intensities could be aligned with the two-hole spatial filter to retain the maximum laser power, along with a simple phase-compensating plate to bring the electric fields across the mode into phase.

### Pulse stretching

A focusing system stretches a pulse width since the focal length of lenses is radius and wavelength dependent due to spherical and chromatic aberrations respectively. For imaging applications which use broadband light source such as white light, the use of an achromatic lens is needed to strongly reduce chromatic aberrations. However, since the bandwidth of a laser source is narrow, laser writing is less affected by chromatic aberrations. On the other hand, lenses with spherical edges present spherical aberrations: the further the rays are from the optical axis, the further is the focal point along the optical axis. Aspheric lenses, where the surface shape deviates from a spherical one, strongly reduce spherical aberrations. However, these are designed for one wavelength and, therefore, spherical aberrations remain for other wavelengths due to the wavelength dependence of the refractive index *n*(*λ*). A few hundred nanometers away from the designed wavelength may route the rays out of the focal point of a few hundred microns, depending on *n*(*λ*) and the NA of the lens, which results in a pulse delay of a few hundred femtoseconds^62^.

Moreover, two other kinds of effect temporally stretch a laser pulse in lenses. First, the pulse front, which is defined as the surface coinciding with the peak of the pulse, moves with group velocity and is thus delayed with respect to the phase front. In a lens, this delay depends on the input radius *r* due to the fact that the path length of the beam is different for various regions of the lens cross-section. This propagation time difference (PTD) is proportional to d*n*/d*λ*. For a singlet lens, the temporal delay Δ*T* due to PDT of a pulse between the middle and the edge of the lens is^24^:

1 $${\rm{\Delta }}T=\frac{{r}_{0}^{2}}{2cf(n-1)}(-\lambda \frac{dn}{d\lambda })$$

Where *r*_0_ is the lens radius, *c* is the speed of light and *f* is the focal length of the lens. Note that d*n*/d*λ* is negative for most transparent materials in the visible and near-infrared ranges.

The second effect is the pulse broadening due to group velocity dispersion (GVD) of the material of the lens. This effect is proportional to d^2^ *n*/d*λ* ^2^ of the material. Since this broadening is also dependent on the path length in the media, the broadening is also dependent on *r*. For a transform-limited pulse (the minimum pulse duration possible for a given optical spectrum), the pulse broadening Δ*τ* due to GVD in a lens is^24^:

2 $$\Delta \tau =\frac{{\lambda }^{3}{r}_{0}^{2}}{4{c}^{2}f(n-1){\tau }_{p}}(\frac{{d}^{2}n}{d{\lambda }^{2}})$$

where *τ*_p_ is the initial pulse duration.

### Glass cover slide method

By placing a glass cover slide in optical contact with the top surface of the sample, the air-glass interface is suppressed and the fs-laser passes through unaffected due to the weak Vander Waals attractive forces established between the atoms of the surfaces which form a temporary direct bond^21^. Therefore, it becomes possible to write waveguides at the very edge of the sample surface. After the writing process, the temporary bond is broken by placing the assembly in water. The cover glass slide falls off, leaving the surface of the sample intact. However, the glass cover slide generates additional spherical aberration. Also, the surfaces must be perfectly flat and clean to meet the Vander Waals conditions, which require a specific technique^63^, and can be very difficult to realize for large glass surfaces.

### Waveguide writing in flexible thin glass sheets using the Dual-Beam technique

Among the different pulse energies in Fig. 5, the best recipe for low loss waveguide was using 1 *μ*J per pulse (Fig. 5j,k,l). The 100 *μ*m thick flexible glass sheet had to be placed using adhesive tapes since vacuum holders curve the sheet by few tens of microns. However, the surface height cannot be perfectly flat, especially for large surfaces. Using the conventional focusing method (Fig. 5j), only few mm long waveguides were achieved without ablation, due to the limited depth of field of 13 *μ*m in which it is possible to write waveguide, and the optical losses could not be measured. Note that surface mapping methods may be used to keep the same relative distance from the surface^29^. Note also that a depth variation of 13 *μ*m in glass is realized by moving the lens vertically by about 9 *μ*m due to the refractive index mismatch, which decreases the precision. Note that this effect is shown in Fig. 3e with a refractive index of 2.4. Using the Dual-Beam technique (Fig. 5l), with a depth of field of 46 *μ*m in which waveguide writing is possible, waveguides were written easily over the full length of the glass sheet (7 cm). Optical loss of 0.7 dB/cm at 1550 nm was measured excluding 0.5 dB coupling loss per facet, using optical fiber butt coupling method and tested on two samples. Since photonics devices have already been fabricated in display screen^18, 19^, these results are of great interest for future flexible display photonics applications.

### Intensity profile measurement of the Dual-Beam technique at the focus

To experimentally ensure that the two beams meet properly at the focus, the intensity profile was measured in air using a scanning slit profiler (see Fig. 3f). The scale of the focus is however not accurate since the scanning slit is not the ideal method to measure a focus profile since the slit edges diffract significantly the light and the sensor sensibility is angle dependent.

### The effective pulse energy density

Since the focal spot shape is elongated using the Dual-Beam technique, an “effective pulse energy density” is used as ×-axis to better compared the data. In fact, with the Dual-Beam technique, the focus volume and a heat dissipation factor (since the elongated focal spot better dissipates the heat and stress) should be added to the pulse energy to represent the ×-axis in Fig. 7c. A focus volume of 80 *μ*m^3^ is obtained from Figs 1b and 6a for the conventional Gaussian focusing method. Therefore, the effective pulse energy density for this method is directly the pulse energy density (pulse energy over 80 *μ*m^3^). The transition from type I to type II (in which the focused laser creates a hole and the waveguide is formed around the focus) should occur at the same effective pulse energy density regardless of the writing method. This phenomenon is used to choose the factor (see Fig. 7c). For the 100 mm/s scan speed and 200 kHz repetition rate recipe, the last type I waveguide (highest energy before type II occurs) using the conventional focusing method was under a pulse energy of 3.5 *μ*J whereas using the Dual-Beam technique it was under a pulse energy of 5 *μ*J, therefore a factor of 0.7 was used to calculate the pulse energy density. For the 10 mm/s and 200 kHz recipe, a factor of 0.67 was chosen, and for the 100 mm/s and 600 kHz recipe, a factor of 0.87 was chosen. The latter factor (closer to 1) was expected since the focal spot shape is less critical under the high repetition rate. Note that multi-scan technique is usually used for better light confinement in type II waveguides and has not been studied yet in this work.
